# Development of Non-Invasive miRNA Markers for Assessing the Quality of Human Induced Pluripotent Stem Cell-Derived Retinal Organoids

**DOI:** 10.3390/ijms25158011

**Published:** 2024-07-23

**Authors:** Hyo Song Park, Ji-Hong Bang, Wook Hyun Jung, Jin Young Yang, Hee Jeong Shin, Ji-Hye Son, Jung Woo Han, Si Hyung Lee, Kyung Hwun Chung, Kyunggon Kim, Hun Soo Chang, Tae Kwann Park

**Affiliations:** 1 Divisions of Ophthalmology, Soonchunhyang University Bucheon Hospital, College of Medicine, Soonchunhyang University, Bucheon 14584, Republic of Korea; 124533@schmc.ac.kr (H.S.P.); 141609@schmc.ac.kr (W.H.J.); 106236@schmc.ac.kr (J.W.H.); sieh12@schmc.ac.kr (S.H.L.); 2 Department of Interdisciplinary Program in Biomedical Science Major, Graduate School, Soonchunhyang University, Asan 31538, Republic of Korea; jihong325@naver.com (J.-H.B.); shj3277@naver.com (H.J.S.); 3 Laboratory of Molecular Therapy for Retinal Degeneration, Soonchunhyang University Bucheon Hospital, Bucheon 31538, Republic of Korea; roswellgirl111@gmail.com (J.Y.Y.); cyung6767@gmail.com (K.H.C.); 4 Department of Microbiology, College of Medicine, Soonchunhyang University, Cheonan 33151, Republic of Korea; son310321@naver.com; 5 Department of Digital Medicine, Brain Korea 21 plus, College of Medicine, University of Ulsan and Asan Medical Center, Seoul 05505, Republic of Korea; kkkon1@amc.seoul.kr

**Keywords:** retinal organoids, exosomes, microRNA, differentiation, non-invasive, markers

## Abstract

Human retinal organoids (ROs) have emerged as valuable tools for studying retinal development, modeling human retinal diseases, and screening drugs. However, their application is limited primarily due to time-intensive generation, high costs, and low reproducibility. Quality assessment of RO differentiation is crucial for their application in research. However, traditional methods such as morphological evaluation and immunohistochemical analysis have limitations due to their lack of precision and invasiveness, respectively. This study aims to identify non-invasive biomarkers for RO differentiation quality using exosomal microRNAs (miRNAs), which are known to reflect cell-specific functions and development in the retina. We differentiated ROs from human induced pluripotent stem cells (hiPSCs) and classified them into ‘superior’ and ‘inferior’ groups based on morphological and immunohistochemical criteria. Exosomes from the conditioned media were isolated and analyzed for miRNA content. Our findings revealed distinct miRNA profiles between superior and inferior ROs, with superior ROs exhibiting higher miRNA diversity and specifically up- or down-regulated miRNAs. Gene ontology and pathway enrichment analyses indicated that the target genes of these miRNAs are involved in neuron proliferation and differentiation. The study suggests the potential of exosomal hsa-miR-654-3p and hsa-miR-451a as non-invasive biomarkers for real-time monitoring of RO quality, facilitating the development of standardized, efficient, and cost-effective culture methods.

## 1. Introduction

Organoids are three-dimensionally organized collections of cells produced through self-renewal and self-organization from pluripotent stem cells [1]. In retinal research, human retinal organoids (ROs) have emerged as valuable tools for studying retinal development, modeling human retinal diseases, and screening drugs to address the limitations of animal models, which exhibit differences in retinal structures and cell components compared to humans [2]. However, the use of ROs in research and industrial applications has been limited primarily due to the time- and cost-intensive nature of their generation and their low reproducibility [3].

Assessment of the quality of retinal organoids (ROs) has been achieved through their morphological characteristics as suggested by Capowski et al. [4], or through immunohistochemistry and gene expression profiling for markers of retinal cell development. The former is non-invasive and easy but may not precisely reflect the biological features of ROs. The latter can provide robust and reliable assessments of RO quality but is inherently destructive. Due to the variance in quality, results from destructive assessments may not be directly comparable across different ROs. Thus, the identification of non-invasive markers for assessing the differentiation quality of ROs, which reflect cell contents and biology, could enable real-time monitoring methods. Such methods are necessary to develop standardized culture methods for ROs that are both time- and cost-effective. However, to date, there have been no studies addressing this need.

Exosomes have attracted increasing attention because they contain various molecular mediators such as proteins, phospholipids, and non-coding RNAs, including microRNAs (miRNAs), that act as messengers during intercellular communication [5]. Various human pluripotent stem cell-derived organoids consistently release extracellular vesicles (EVs), including exosomes [6,7]. It has been reported that target genes of miRNAs in EVs released by human induced pluripotent stem cell (hiPSC)-derived ROs are involved in retinal development, ganglion cell differentiation, and photoreceptor function [7]. Additionally, it is known that miRNAs present in eye tissue exhibit tissue- and cell-specificity, such as corneal epithelium, lens, and retina [8]. In fact, the miR-183/96/182 cluster genes have been reported to play a crucial role in retinal tissue morphogenesis and in the transdifferentiation of human retinal pigment epithelial cells into retinal neurons by regulating important genes in retina development and function, such as PAX6, CCND2, CDK5R1, and CCNJ [9,10]. Additionally, a set of miRNAs in light-responsive human retinal organoids was differentially regulated by distinct wavelengths of light and had a rapid turnover [11]. These observations suggest that various miRNAs could play specific roles in the development and functions of cells in the retina [12].

In this study, we categorized 60-day-cultured retinal organoids (ROs) into ‘good quality’ (called “superior”) and ‘poor quality’ (called “inferior”). The superior organoids were defined by laminated retinal ganglion cell layers and well-structured neuroblastic layers at the stage of differentiation. These layers are hallmarks of producing useful ROs with highly organized structures, such as inner and outer nuclear layers and plexiform layers, with functional photoreceptors at a later stage that resemble the function of the human retina. We then analyzed the exosomal miRNA landscapes in conditioned media of ROs of varying quality to identify miRNAs associated with superior and inferior differentiation processes. Furthermore, we investigated the potential role of exosomal miRNAs in regulating RO development and function. Our findings can provide new insights into the roles and mechanisms of exosomal miRNAs in RO differentiation, emphasizing their potential as non-invasive biomarkers for identifying higher quality ROs.

## 2. Results

### 2.1. Classification of the Superior and Inferior Retinal Organoids

**Figure 1:**
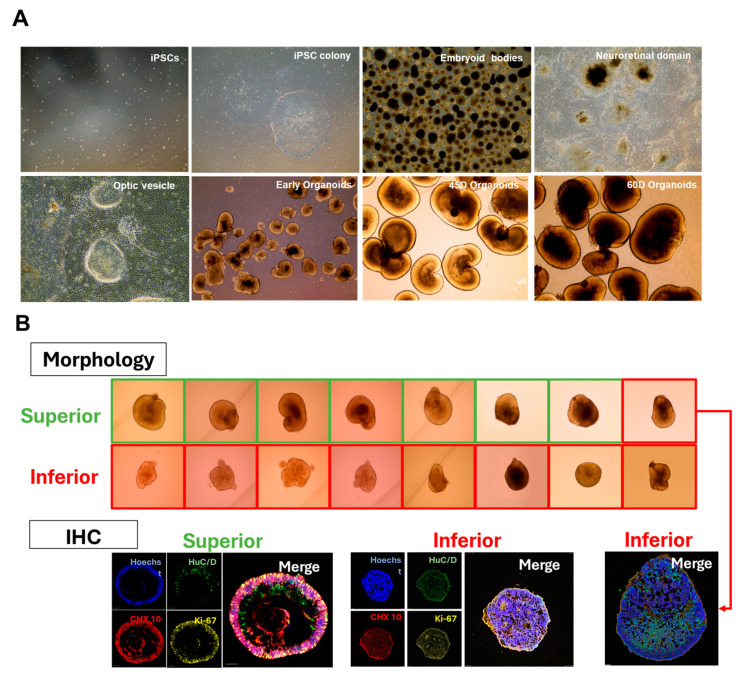
(**A**) Main steps of hiPSC-derived RO development in vitro: hiPSC colony, embryoid body formation, optic vesicles, and ROs. (**B**) Determination of quality of ROs based on their morphology (upper panel) and immunohistochemistry (IHC, lower panel). In morphological determination, ROs with a reduced or absent phase-bright outer rim which appeared in early culture and with a phase-dark core were defined as good (‘superior’)-quality ROs, while those lacking these characteristics were defined as bad (‘inferior’)-quality. Immunofluorescence staining of human HuC/D protein (green) in retinal ganglion cells and CHX10 (red) and Ki-67 (yellow) in retinal progenitors at D60 of differentiation (scale bar = 20 μm). Eight ROs from each quality group were selected for subsequent analysis. One RO categorized as ‘superior’ based on its morphology was subsequently reclassified as ‘inferior’ due to the absence of laminated expression of the protein markers (lower right panel).

Three-dimensional (3D) ROs were generated using hiPSCs, as presented in Figure 1A. The hiPSC-derived aggregates were progressively formed from embryoid bodies (EBs). Optic vesicles were manually isolated between days 25 to 28 and subsequently formed 3D ROs when cultured in suspension. After 28 days of differentiation, the ROs exhibited distinctive morphological and phenotypic characteristics, manifesting a bright, stratified layer towards the periphery. We then successfully cultured and maintained the ROs for up to 60 days (Figure 1A).

At day 55, the ROs were categorized into ‘superior’ and ‘inferior’ based on their microscopic morphological features as described in Methods. Among the ROs classified as superior, seven exhibited layered expression of HuC/D, CHX10, and Ki-67 in their periphery, as observed in the IHC finding. However, one RO initially categorized as ‘superior’ was subsequently reclassified as ‘inferior’ due to the absence of laminated expression of the protein markers (Figure 1B). The IHC-based definition was used for the following DEG analysis for exosomal miRNAs.

### 2.2. Exosome Isolation and Diversity of Exosomal miRNAs

**Figure 2:**
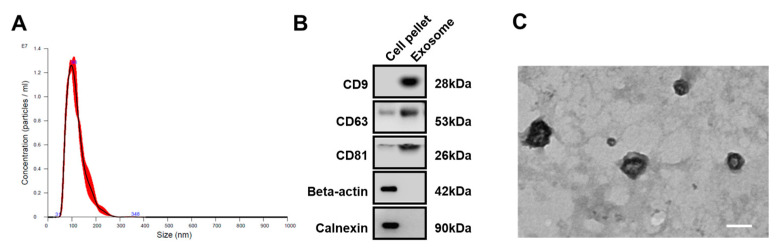
Evaluation of isolated exosomes from conditioned media of hiPSC-derived ROs. (**A**) Diameter and concentration distribution of exosomes from hiPSC-derived RO conditioned media at D60. (**B**) Western blot analysis of the expression of the exosomal markers (CD63, CD81, and CD9) and cytoplasmic markers (beta-actin and calnexin) in exosomes from hiPSC-derived ROs. (**C**) Transmission electron microscopy images of exosomes from hiPSC-derived ROs (scale bar = 100 μm).

Exosomes from the conditioned medium of hiPSC-derived ROs at day 55~65 were successfully isolated and analyzed for their size distribution using NTA, which showed an average diameter of 117.3 ± 1.8 nm, with a peak diameter of 104.1 ± 4.5 nm (Figure 2A). Western blotting for exosomal markers [13], including CD9, CD63, and CD81, showed stronger band intensities for isolated exosomes compared to the crude precipitates of the RO-conditioned medium (Figure 2B), while β-actin and calnexin, intracellular proteins known to be negative markers for exosomes [13], were not detected in the isolated exosomes. Examination with TEM revealed round, cup-shaped exosomes with an average diameter of approximately 110 nm (Figure 2C).

**Figure 3:**
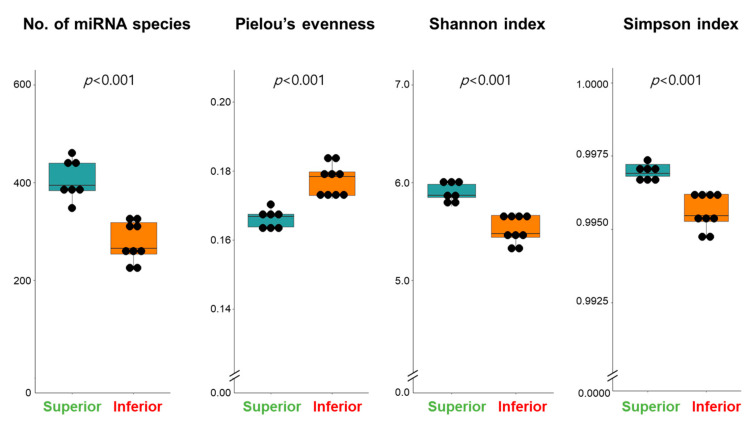
The richness, evenness, and alpha diversity of exosomal miRNA in conditioned media of superior (*n* = 7) and inferior (*n* = 9) ROs at D60.

A total of 617 mature miRNA species were detected in exosomes isolated from the conditioned media of 16 ROs. We hypothesized that properly differentiated ROs (superior ROs) would exhibit a higher diversity of secreted exosomal miRNAs compared to poorly differentiated ROs (inferior ROs) due to their relatively heterogeneous cell components. Thus, the alpha diversity of expressed exosomal miRNAs was compared between the superior and inferior ROs. The number of expressed miRNA species was significantly higher in exosomes produced by superior ROs than that of inferior ROs (406.8 ± 15.4 vs. 278.0 ± 13.7, *p* < 0.05, Figure 3). The evenness among miRNA species was lower in superior ROs than in inferior ROs (0.167 ± 0.001 vs. 0.177 ± 0.002, *p* < 0.05). The alpha diversity measured by both the Shannon and Simpson index was higher in superior ROs than in inferior ROs (Shannon index, 5.90 ± 0.04 vs. 5.52 ± 0.05; Simpson index, 0.9970 ± 0.0001 vs. 0.9956 ± 0.0002, *p* < 0.05).

### 2.3. Differentially Expressed Exosomal miRNAs between Superior and Inferior ROs

**Figure 4:**
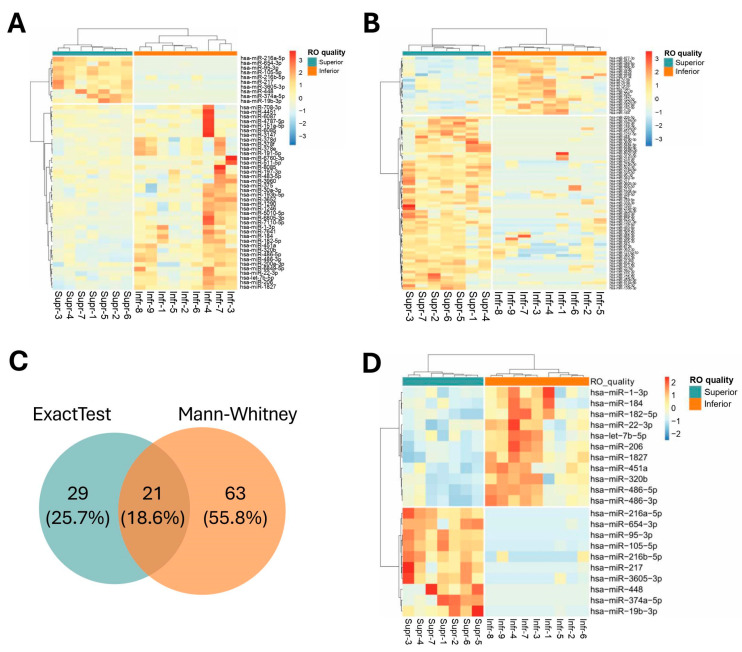
Differentially expressed (DE) miRNA analysis and identification of exosomal miRNAs associated with quality of ROs. (**A**) Expression levels of DE miRNAs identified as significant in the ExactTest of the edgeR package (FDR *q* < 0.05). (**B**) Expression levels of DE miRNAs identified as significant in the non-parametric Mann–Whitney’s U test (*p* < 0.05). (**C**) Venn diagram of the number of DE miRNAs in both statistical analyses. (**D**) Heatmap of common miRNAs in both analyses.

To identify specific exosomal miRNAs produced by superior ROs, a DEG analysis was conducted. In the exact test, 10 miRNAs were found to be upregulated, while 40 miRNAs were downregulated in the exosomes derived from superior ROs compared to those from inferior ROs (FDR *q* < 0.05, Figure 4A and Supplementary Table S1). Next, a non-parametric Mann–Whitney’s U test was conducted to identify robust markers for assessing the quality of ROs. The results showed that 63 genes were upregulated, and 21 miRNAs were downregulated in the exosomes derived from superior ROs compared to those from inferior ROs. (*p* < 0.05, Figure 4B and Supplementary Table S2). When comparing the results of the exact test and Mann–Whitney’s U test to identify promising markers for distinguishing superior ROs from inferior ROs, 21 miRNAs were found common to both analyses, with 10 upregulated miRNAs and 11 downregulated miRNAs (Figure 4C,D and Supplementary Table S3). 

**Figure 5:**
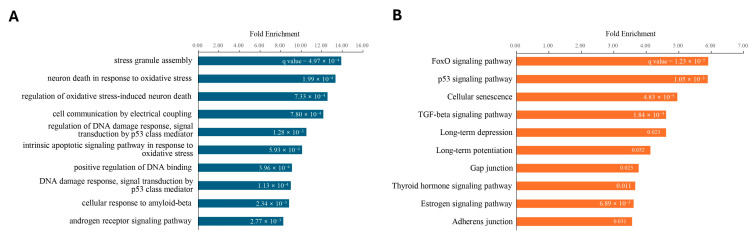
(**A**) Biological processes in gene ontology and (**B**) KEGG pathway enrichment of 305 genes targeted by 10 upregulated exosomal miRNAs in superior ROs. Fold enrichment represents the observed ratio of genes to the 305 query genes divided by the expected ratio of genes to the reference gene set. The number in each bar indicates the FDR *q*-value of enrichment.

Out of the 10 upregulated exosomal miRNAs in superior ROs, 5550 target genes were estimated (Supplementary Table S4). Among these, 4951 genes were detected in the Gene Expression Omnibus dataset, which was a single-cell RNA-seq study for ROs at day 45 and 80 (GSE181737) [14]. Out of these, 305 genes were targeted by more than three of the ten upregulated exosomal miRNAs (Supplementary Table S5). Gene ontology enrichment analysis revealed that the most enriched biological process of the 305 genes were “stress granule assembly”, “neuron death in response to oxidative stress”, “regulation of oxidative stress-induced neuron death”, “cell communication by electrical coupling”, and “regulation of DNA damage response, signal transduction by p53 class mediator” (Figure 5A and Supplementary Table S6). In pathway enrichment analysis, the 305 target genes were enriched in “FoxO signaling pathway”, “p53 signaling pathway”, “Cellular senescence”, “TGF-beta signaling pathway”, and “Long-term depression” (Figure 5B and Supplementary Table S7).

**Figure 6:**
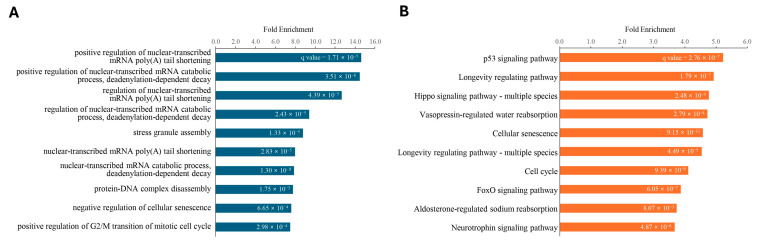
(**A**) Biological processes in gene ontology and (**B**) KEGG pathway enrichment of 736 genes targeted by 11 downregulated exosomal miRNAs in superior ROs. Fold enrichment represents the observed ratio of genes to the 736 query genes divided by the expected ratio of genes to the reference gene set. The number in each bar indicates the FDR *q*-value of enrichment.

In contrast, out of the 11 downregulated exosomal miRNAs in superior ROs, 10,822 target genes were estimated (Supplementary Table S8). Among these, 9401 genes were detected in the single cell RNA-seq study for ROs at day 45 and 80. Of these, 736 genes were targeted by more than three of the eleven downregulated exosomal miRNAs (Supplementary Table S9). Gene ontology enrichment analysis revealed that these 736 genes were predominantly associated with the biological process term in “positive regulation of nuclear-transcribed mRNA poly(A) tail shortening”, “positive regulation of nuclear-transcribed mRNA catabolic process, deadenylation-dependent decay”, “regulation of nuclear-transcribed mRNA poly(A) tail shortening”, “RISC complex assembly”, and “progesterone receptor signaling pathway” (Figure 6A and Supplementary Table S10). In pathway enrichment analysis, the 736 target genes were enriched in “p53 signaling pathway”, “Longevity regulating pathway”, “Hippo signaling pathway”, “Vasopressin-regulated water reabsorption”, and “Cellular senescence” (Figure 6B and Supplementary Table S11).

### 2.4. Identification of Representative miRNA Markers for Superior ROs

**Figure 7:**
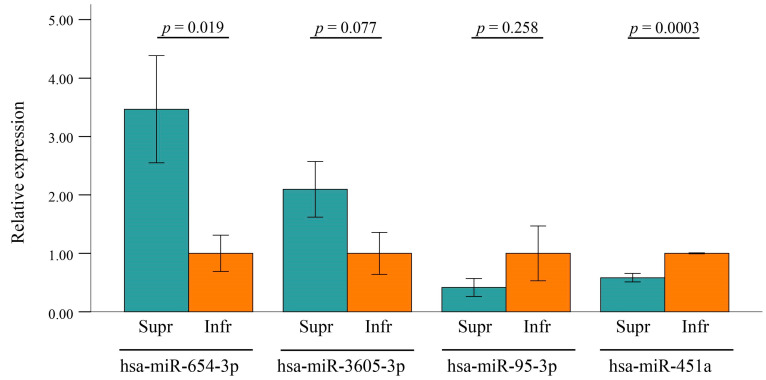
Result of quantitative real-time PCR for differentially expressed exosomal miRNAs. Relative expression represents the ratio of 2^−ΔΔCt^ value of miRNAs to the average 2^−ΔΔCt^ value of corresponding miRNAs in inferior ROs. *p* values were obtained using Student’s *t*-test.

To validate the potential of miRNA expression levels as markers for superior ROs, quantitative real-time PCR was conducted on the three miRNAs using additional exosome samples isolated from conditioned media of independent superior and inferior ROs, distinct from the 16 samples used for NGS. In addition to the three miRNAs, hsa-miR-3605-3p and hsa-miR-451a were included in the qPCR analysis as they were considered potential positive and negative markers, respectively, based on the DEG analysis (see Figure 4D). The results indicated that the level of hsa-miR-654-3p in superior ROs was 3.47 ± 0.92 times higher than that in inferior ROs (*p* = 0.019, see Figure 7). Hsa-miR-3605-3p showed a trend towards a 2.10 ± 0.48-fold increase in superior ROs compared to inferior ROs (*p* = 0.077). However, the level of hsa-miR-95-3p did not show a statistically significant difference between the two types of ROs (*p* = 0.258). Notably, the level of hsa-miR-451a, a potential negative marker, was significantly lower in superior ROs compared to inferior ROs (fold change = 0.58 ± 0.07, *p* = 0.0003). Hsa-miR-320b was not detected in the qPCR analysis.

## 3. Discussion

Our study indicates that well-differentiated superior ROs show a more diverse landscape of exosomal miRNAs. By comparing exosomal miRNAs of superior and inferior ROs, we identified 10 upregulated and 11 downregulated miRNAs. Target genes of up-regulated miRNAs in superior ROs were mainly associated with neuronal cell death in response to oxidative stress. In contrast, target genes of down-regulated miRNAs in superior ROs were associated with mRNA catabolic processes. Among some of the miRNAs which exhibited significant differences in the expression levels according to RO quality, hsa-miR-654-3p and hsa-miR-451a emerged as potential markers for RO grading. Specifically, hsa-miR-654-3p was expressed more and hsa-miR-451a less in superior ROs than in inferior ROs.

After Nakano et al. reported the self-organization of optic cups and stratified neural retinas from human ESCs [15], the protocol for RO generation has advanced significantly over the past two decades [16,17,18,19]. However, large-scale RO production remains limited due to low yield and reproducibility [3,20]. Consequently, the application of ROs in retina research and industrial fields is restricted. Although morphological features at the developmental stage provide some insights, solid quality assurance largely depends on evaluating the protein and gene expression of retinal markers, which is inevitably destructive. In our study, we observed that the morphological assessment of RO quality did not consistently correlate with immunohistochemical observations of HuC/D, CHX10, and Ki67, well-known markers for RO development at D60. Notably, one of eight ROs deemed superior based on morphological features did not exhibit structured and laminated characteristics in its marker protein expressions (Figure 1B). To address this issue, we evaluated exosomal miRNAs released into the culture medium from ROs which were specifically expressed in superior and inferior ROs. This approach could be useful for non-invasive evaluation of RO quality.

Until recently, there have been only a few reports regarding miRNAs in relation to RO differentiation and retinal cell functions. It has been previously established that the miR-183/96/182 cluster (hsa-miR-182-5p, hsa-miR-183-5p, hsa-miR-96-5p) is an important morphogenetic factor targeting PAX6 expression in differentiating human ROs [9]. The cluster is known to be highly expressed in pluripotent stem cells and sensory organs, including the retina, and is gradually downregulated during the initial steps of hiPSC differentiation into ROs, reaching minimal expression around D55 [9]. Recently, Celiker et al. identified a subset of light-regulated miRNAs in ROs using a photostimulation device, which were differentially and rapidly regulated by distinct wavelengths of light [11]. However, in our study, the reported functional miRNAs were not detected, which may be due to intrinsic differences between the miRNA components of the ROs themselves and those of extracellular vesicles [7].

In context of exosomes, Zhou et al. reported that extracellular vesicles released by human ROs carry small noncoding RNAs which targeted genes and pathways involved in the mechanisms of retinogenesis relevant to specific developmental stages corresponding to the hallmarks of native human retina development [7]. They also revealed that hsa-miR-6748-3p, hsa-miR-4488, and hsa-miR-4516 were some of the abundant miRNAs in extracellular vesicles of ROs at D63. In our observation, although these miRNAs were detected in exosomes, their expression levels were comparable between superior and inferior ROs, which suggests that these miRNAs are likely involved in common pathways of RO development, regardless of their differentiation quality.

In our observation, specifically up- and down-regulated exosomal miRNAs in superior RO compared to those in inferior RO showed reliable biological characteristics reflecting the differentiation stage of RO. The target genes of upregulated exosomal miRNAs in superior ROs were primarily involved in neuronal death caused by oxidative stress and in response to DNA damage, suggesting superior ROs may inhibit these cellular processes compared to inferior ROs. The results support our previous findings, in which RO-derived exosome treatment reduced photoreceptor apoptosis, prevented outer nuclear layer thinning, and preserved visual function in the Royal College of Surgeons (RCS) rats, and rescued retinal pigment epithelium (RPE) cells from apoptosis induced by oxidative stress [21]. Inversely, target genes of downregulated exosomal miRNAs in superior ROs were involved in mRNA catabolism mediated by miRNAs. Rapid degradation of mRNA has been known to be associated with exuberant cellular responses to stimuli [22] and with the cell cycle [23], suggesting that superior ROs may exert dynamic turnover of mRNA required for retinal cell differentiation and proliferation. Thus, our observations support the possible roles of miRNAs in RO development and their usefulness as markers for assessing the quality of ROs.

In this study, hsa-miR-654-3p and hsa-miR-451a were regarded as potential biomarkers to evaluate the quality of differentiation in ROs. hsa-miR-654-3p is known to be downregulated in many types of malignant tumors, including hepatocellular carcinoma, non-small cell lung cancer, metastatic prostate cancer, and papillary thyroid cancer, suggesting its role as a tumor suppressor [24,25,26,27]. Regarding the eye, hsa-miR-654-3p was included in top 15 upregulated miRNAs in the aqueous humor of patients with polypoidal choroidal vasculopathy [28]. Hsa-miR-451a is also one of the biomarkers proven to be down-regulated in many human malignancies and is correlated with tumor progression. The function of hsa-miR-451a ranges from regulating the proliferation and migration of cells to inhibiting epithelial-mesenchymal transition in many diseases [29,30]. It is also known that hsa-miR-451a inhibits hepatic gluconeogenesis and alleviates hyperglycemia, and its expression is regulated by glucose, lipids, and hypoxia [30,31,32,33]. Additionally, it was reported to regulate RPE function by promoting mitochondrial function in proliferative diabetic retinopathy and was also found to be upregulated in the aqueous humors of patients with primary open-angle glaucoma [34,35]. Regarding the roles of exosomes in intercellular communications, the functions of hsa-miR-654-3p and hsa-miR-451a in cell-cell interactions during retinal development should be evaluated in future study.

In conclusion, this study has described the exosomal miRNA expression profile in relation to the differentiation quality of RO for the first time to the best of our knowledge. Our study can provide new insights into the roles of exosomal miRNAs in the differentiation process of ROs. Additionally, hsa-miR-654-3p and hsa-miR-451a show promise as potential biomarkers for noninvasive quality assessment of RO differentiation, which is crucial for the advancement and application of ROs.

## 4. Materials and Methods

### 4.1. hiPSC Cultures

The American Type Culture Collection (ATCC) DYR0100 hiPSCs (ACS-1011; ATCC, Manassas, VA, USA) were used in our experiments. The hiPSCs were maintained in Essential 8 (E8) medium (GibcoTM; Thermo Fisher Scientific, Waltham, MA, USA) on culture dishes coated with vitronectin (Thermo Fisher Scientific). The cells were routinely cultured at 37 °C in a standard 5% CO_2_/95% air incubator with daily medium changes. Upon reaching approximately 70% to 80% confluency, the cells were mechanically passaged with the enzyme-free reagent ReLeSR (StemCell Technologies, Vancouver, BC, Canada) every 5 to 7 days. After that, the detached cell aggregates were collected in an E8 medium and carefully pipetted up and down to obtain a uniform suspension of cell aggregates, which were related at 1/10 to 1/60, depending on the confluence.

### 4.2. Differentiation into Three-Dimensional ROs and Conditioned Media Sample Collection

The ROs were differentiated from ATCC DYR0100 hiPSCs according to a retinal differentiation protocol recently described by Lee et al. [36]. The hiPSCs were maintained on vitronectin-coated culture dishes with E8 medium (Thermo Fisher Scientific) and dissociated by treatment with ReLeSR (StemCell Technologies). Next, the dissociated cells were plated on a low-attachment 6-well plate containing E8 medium (Thermo Fisher Scientific) with 3 µM ROCK inhibitor Y27632 (Tocris Biosciences, Abingdon, UK) and 3 µM Blebbistatin (Tocris Biosciences) at day 0 to induce embryoid body (EB) formation. Subsequently, the EBs were gradually replaced with neural induction medium (NIM) containing Dulbecco’s Modified Eagle Medium/Nutrient Mixture F-12 (DMEM/F12; 1:1; Thermo Fisher Scientific), 1% N-2 supplement (Thermo Fisher Scientific), non-essential amino acids (NEAAs), and 2 µg/mL heparin (StemCell Technologies) from E8 medium without ROCK inhibitor Y27632 and Blebbistatin (Tocris Biosciences). The day of detachment was annotated as day 0, with the medium being changed on days 1 (25% NIM), 2 (50% NIM), and 3 (100% NIM). On day 7, the EBs were plated on 35 mm Matrigel-coated dishes (Corning Life Sciences, Tewksbury, MA, USA) containing NIM at a density of 150 EB per dish. On day 15, the medium was switched from NIM to retinal differentiation medium (RDM), consisting of DMEM/F12 (3:1), 2% B-27 supplement without vitamin A (Thermo Fisher Scientific), NEAAs, and antibiotic-antimycotic solution (Thermo Fisher Scientific), and was changed every other day. On days 25 to 28, loosely adherent central portions of the neural clusters were lifted using a P1000 pipette under an Evos XL cell imaging microscope (Invitrogen, Waltham, MA, USA). The selected optic vesicle-like aggregates were further cultured to form three-dimensional (3D) ROs with RDM, supplemented with 10% exosome-depleted fetal bovine serum (FBS; Cat. No. A2720801; Thermo Fisher Scientific), 100 mM taurine (Sigma-Aldrich, St. Louis, MO, USA), and 2 mM L-alanyl-L-glutamine dipeptide (GlutaMAXTM; Thermo Fisher Scientific). For long-term organoid culture, the medium was changed every 3 days until day 55.

### 4.3. Morphological and Immunohistochemical Quality Assessment of iPSC-ROs

On day 55, quality of ROs was assessed based on morphological features suggested by Capowski et al. [4,37]. Briefly, ROs displaying a reduced or absent phase-bright outer rim, which typically appears in early culture, along with a phase-dark core were defined as a good quality (labeled ‘superior’). Conversely, ROs lacking these characteristics were defined as bad quality (labeled ‘inferior’). Following selection, eight superior and eight inferior ROs were individually transferred into 96-well culture plate and cultured for additional 10 days. Conditioned media were collected every 2 days and pooled for subsequent exosome isolation and small RNA sequencing. Approximately 1 mL of medium was collected for each set of 16 ROs. The collected media were centrifuged at 3000× *g* for 10 min to remove cell debris and stored at −80 °C until further analysis.

At day 65, following the media collection, the quality of each ROs was reassessed by immunohistochemical (IHC) staining. Each RO was fixed in 4% paraformaldehyde at room temperature for 30 min and cryoprotected in 15% sucrose for at least 2 h, followed by 30% sucrose overnight, before being embedded in optimum cutting temperature (OCT) compound. The OCT blocks were sectioned at 10 μm thickness and incubated at room temperature for at least 1 h, before immunostaining or storage at −80 °C. Slides were rinsed in 0.1% Triton X-100 (Sigma-Aldrich, St. Louis, MO, USA) in PBS and blocked with 5% donkey serum in PBS for 2 h, and incubated overnight at 4 °C with primary antibodies against HuC/D, CHX10 and Ki-67 diluted in blocking solution. Species-specific secondary antibodies, conjugated with Alexa Fluor 488 or 568, were diluted in antibody dilution buffer and incubated at room temperature for 2 h. Hoechst^®^ 33342 (H1399, Invitrogen) was used for nuclear staining. Fluorescence images were captured using a confocal microscope (DMI8; Leica Camera, Wetzlar, Germany). ROs displaying a well-laminated layer stained with CHX10 and Ki-67 and a sublayer stained with HuC/D were defined as superior ROs, while ROs lacking these features were categorized as the inferior one. The study scheme is depicted in Supplementary Figure S1.

### 4.4. Exosome Isolation and Characterization

Exosomes were extracted from the supernatant of the culture medium using a miRCURY exosome isolation kit (Qiagen, Hilden, Germany) according to the manufacturer’s instructions. In essence, an initial spin was performed at 3000× *g* for 10 min to remove cells and debris, then the medium was filtered. The corresponding amounts of reagents were added proportional to the organoid medium volume. The mixtures were vortexed and incubated at 4 °C overnight, and then centrifuged at 10,000× *g* for 30 min at 20 °C, followed by exosome pellet resuspension in the manufacturer-supplied suspension buffer. The exosome pellets were resuspended in a 50 μL suspension buffer each with 1 mL starting volumes. All exosomes were stored at −80 °C immediately after isolation until further experimentation.

Nanoparticle tracking analysis (NTA) was performed to determine the concentration and size of organoid exosomes [38]. The exosomes were characterized using a NanoSight NS300 instrument equipped with NTA 3.4 analytical software and a 488-nm laser (Malvern Panalytical Ltd., Malvern, UK); at least five 30 s videos were recorded per sample in light scatter mode at a camera level of 11 to 13. The software settings for the analysis were kept constants for all measurements (screen gain = 10, detection threshold = 7). The exosomes were diluted in 0.22 µM filtered phosphate-buffered saline (PBS) to an appropriate concentration before analysis.

Exosomes derived from ROs were morphologically analyzed by transmission electron microscopy [39]. An exosome suspension diluted in PBS at a ratio of 1:10 was incubated on a 200-mesh sized, formvar/carbon-coated and charged nickel grid (Electron Microscopy Sciences, Hatfield, PA, USA) for 2 min. Next, the grid was fixed in 2.5% glutaraldehyde for 10 min and then washed thrice with 0.1 M PBS, followed by a brief contact of its surface with a water droplet. The grid was blotted to remove excess liquid and was then placed in 8 µL of 2% uranyl acetate for 2 min; it was then subsequently examined and imaged using a JEM-1400 flash electron microscope equipped with an AMT XR401 scientific complementary metal-oxide-semiconductor (sCMOS) camera and AMT Capture Engine software version 7.00 (JEOL Ltd., Tokyo, Japan).

The Western blot was used to determine the exosomal protein markers [40]. The exosomal proteins (5 μg/well) were loaded and electrophoresed on 10% polyacrylamide gels; then transferred to nitrocellulose membranes at 80 V for 120 min. The membranes were blocked for 1 h at room temperature (RT) in 5% skim milk and washed in 0.1% Tween-20 in Tris-buffered saline (TBS), followed by incubation overnight at 4 °C with an anti-CD9 monoclonal antibody 1:500 dilution (Cell Signaling Technology, Danvers, MA, USA), anti-CD63 monoclonal antibody 1:1000 dilution (Santa Cruz Biotechnology, Dallas, TX, USA), anti-CD81 monoclonal antibody 1:500 dilution (Santa Cruz Biotechnology), anti-Calnexin monoclonal antibody 1:500 dilution (Cell Signaling Technology), and anti-β-actin monoclonal antibody 1:10,000 dilution (Sigma-Aldrich). The membranes were then incubated for 1 h at RT with a horseradish peroxidase-conjugated secondary antibody 1:5000 dilution (GenDEPOT, Baker, TX, USA). The target protein was detected by an enhanced chemiluminescence solution (Amersham Pharmacia Biotech, Buckinghamshire, UK) and visualized using the digital system Azure Biosystems C280 (Azure biosystems, Dublin, CA, USA).

### 4.5. Small RNA Sequencing and Gene Ontology-Pathway Enrichment Analysis

The exosomal RNAs were extracted using Trizol reagent (Invitrogen) and TRIzol LS reagent (Ambion, Carlsbad, CA, USA) respectively, according to the manufacturer’s instructions. RNA quality was assessed by Agilent 2100 bioanalyzer using the RNA 6000 Pico Chip (Agilent Technologies, Amstelveen, The Netherlands), and RNA quantification was performed using a NanoDrop 2000 Spectrophotometer system (Thermo Fisher Scientific). The construction of a miRNA library was performed using the NEBNext Multiplex Small RNA Library Prep kit (New England BioLabs, Ipswich, MA, USA) according to the manufacturer’s instructions. Briefly, for the library construction, total RNA from each sample was used to ligate the adaptors and then cDNA was synthesized using reverse-transcriptase with adaptor-specific primers. PCR was performed for library amplification and the libraries were cleaned up using QIAquick PCR Purification Kit (Qiagen) and the PAGE gel. The yield and size distribution of the small RNA libraries were assessed by the Agilent 2100 Bioanalyzer instrument for the High-sensitivity DNA Assay (Agilent Technologies). High-throughput sequences were produced by NextSeq500 system as a way of single-end 75 sequencing (Illumina, Inc., San Diego, CA, USA). The data analysis sequence reads were mapped by bowtie2 software tool (http://bowtie-bio.sourceforge.net/bowtie2/index.shtml, accessed on 14 March 2024) to obtain a bam file. The mature miRNA sequence was used as a reference for mapping and the read counts mapped on the mature miRNA sequences were extracted from the alignment file using bedtools version 2.25.0 (Quinlan and Hall, 2010) [41] and Bioconductor [42] that uses R statistical programming language (R development Core Team, 2016). The read counts were used to determine the expression level of miRNAs. The data was processed based on the count per million (CPM)+. The trimmed mean of the M-values (TMM) normalization method, and differentially expressed gene (DEG) analysis was conducted using an exactTest function using EdgeR within R [43] and a Mann–Whitney’s U test within SPSS SPSS software (version 24.0; SPSS Inc., Chicago, IL, USA). pheatmap packages (version 1.0.12) were used to visualize the results of DEG analysis.

The target genes of miRNAs were predicted using miRTarBase version 8.0 and TarBase v8.0 in miRNet (https://www.mirnet.ca, accessed on 14 March 2024). We exclude target genes which were not detected (average CPM < 1) in a single cell RNA-seq data for retinal organoid at day 45 and 80 (GSE181737) [14]. Gene ontology and KEGG pathway enrichment analyses for the target genes were performed employing the clusterProfiler [44]. The similarities between the obtained Gene ontology or KEGG pathways were calculated and visualized using the enrichplot package (version 1.23.2).

### 4.6. miRNA qRT-PCR Analysis

During miRNA-specific reverse transcription, miRNA was reverse-transcribed to cDNA using the TaqMan^®^ MicroRNA Reverse Transcription Kit (Applied Biosystems, Waltham, MA, USA) and specific primers according to the manufacturer’s instructions. Reverse transcription was performed as follows: 30 min at 16 °C, 30 min at 42 °C, followed by 5 min at 85 °C. The resulting cDNAs were stored at –80 °C until use.

qRT-PCR was used to quantify the expression levels of mature miRNAs using TaqMan^®^ MicroRNA Assay kits and the TaqMan^®^ Universal Master Mix (Applied Biosystems) with the aid of an ABI 7500 real-time PCR system according to the manufacturer’s instructions. miR-30e-5p and miR-151a-3p served as endogenous controls when normalizing the expression levels of target miRNAs. The relative quantification (Rq) of each miRNA expression level was calculated using the 2^−ΔΔCT^ threshold cycle method. qRT-PCR was performed as follows: 2 min at 50 °C, 10 min at 95 °C, followed by 40 cycles at 95 °C for 15 s and 60 °C for 1 min. The statistical significance of the differences was analyzed by the Mann–Whitney U-test using SPSS software (version 24.0; SPSS Inc.), and the significance threshold was set at a *p*-value < 0.05.

## Data Availability

Data will be made available on request.

## Supplementary Materials

The following supporting information can be downloaded at: https://www.mdpi.com/article/10.3390/ijms25158011/s1.
