# Supplementary material for: Development of Non-Invasive miRNA Markers for Assessing the Quality of Human Induced Pluripotent Stem Cell-Derived Retinal Organoids
Source: Int J Mol Sci. 2024 Jul 23;25(15):8011. doi: 10.3390/ijms25158011 (PMC11312389; doi:10.3390/ijms25158011)
Supplement: Supplementary file 1 [file ijms-25-08011-s001.zip › Supplementary FigureS1.pdf]

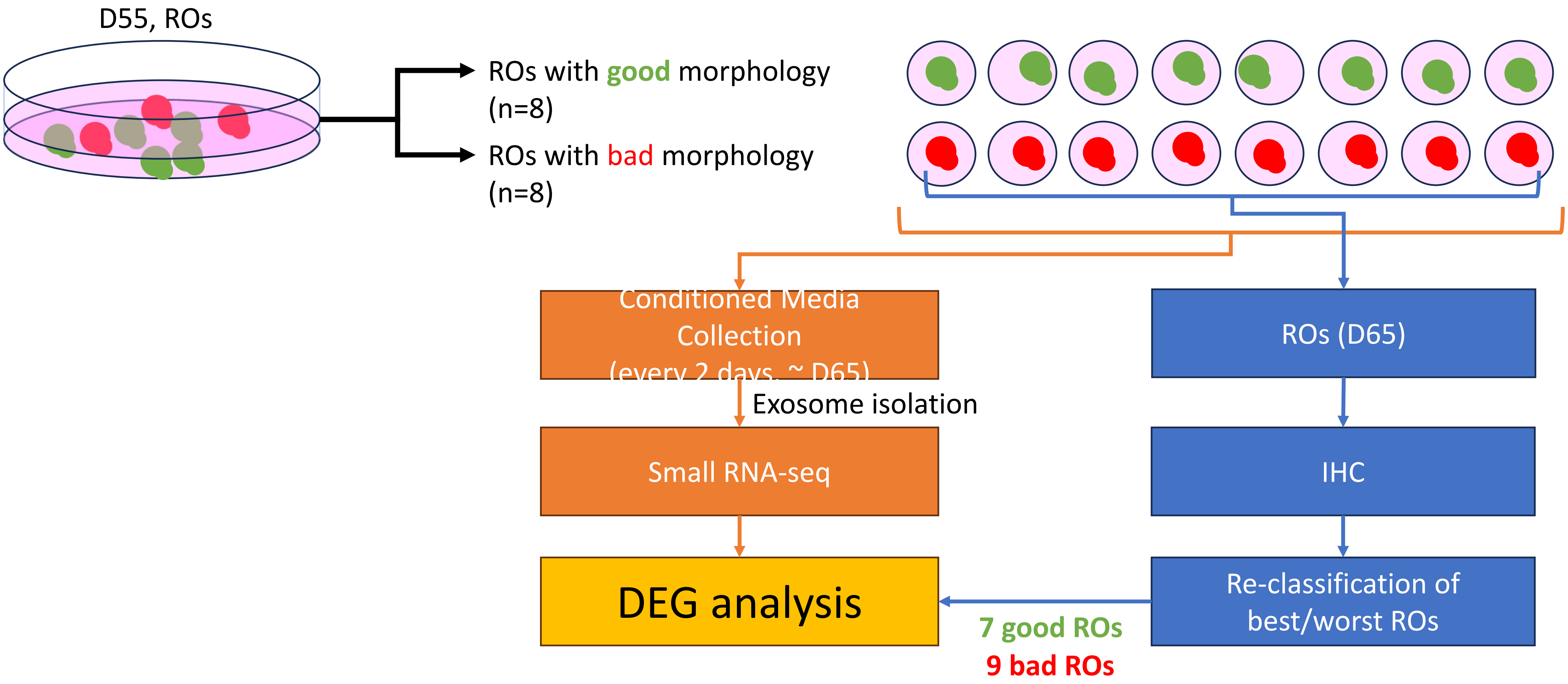

Supplementary Figure S1. The scheme of this study. On day 55, the quality of retinal organoids (ROs) was assessed based on morphological features as suggested by Capowski et al. [4,37]. Following selection, eight superior (good) and eight inferior (bad) ROs were transferred into a 96-well culture plate and cultured for an additional 10 days. The conditioned medium was collected every 2 days and pooled for subsequent small RNA sequencing. On day 65, the quality of each RO was reassessed using immunohistochemical (IHC) staining for proteins of retinal ganglion cells (HuC/D) and retinal progenitor cells (CHX10 and Ki-67). ROs exhibiting well-laminated layers stained with CHX10 and Ki-67, as well as sublayers stained with HuC/D, were classified as 'superior', while those lacking these features were classified as 'inferior'. Following reassessment, differentially expressed miRNA analysis was conducted.
